# A pathogenic IFNα, BLyS and IL-17 axis in Systemic Lupus Erythematosus patients

**DOI:** 10.1038/srep20651

**Published:** 2016-02-05

**Authors:** Patricia López, Javier Rodríguez-Carrio, Luis Caminal-Montero, Lourdes Mozo, Ana Suárez

**Affiliations:** 1 Department of Functional Biology, Immunology Area, Faculty of Medicine, University of Oviedo, Oviedo, 33006, Spain; 2 Department of Internal Medicine, Hospital Universitario Central de Asturias, Oviedo, 33011, Spain; 3 Department of Immunology, Hospital Universitario Central de Asturias, Oviedo, 33011, Spain

## Abstract

This study aims to analyze in depth the role of IFNα in the upregulation of BLyS in different leukocyte populations and the possible relationship of these molecules with IL-17 and other pathogenic cytokines in SLE. Thus, IFNAR1 and membrane BLyS (mBLyS) expression was upregulated on various blood cell types from patients and closely correlated in all individuals. Moreover, BLyS serum levels associated positively with IFNα and IL-17A amounts, as well as with mBLyS on B cells and neutrophils. Interestingly, mBLyS on neutrophils was also correlated with IL-17A levels. Additionally, intracellular IL-17A expression was increased in both CD4^+^ lymphocytes and neutrophils from patients, and IL-17^+^CD4^+^ T cell frequency was associated with serum IFNα and IFNRA1 expression on B cells. Finally, *in vitro* assays support an IFNα role in the activation of Th17 cells in SLE. In conclusion, these data suggest that IFNα, BLyS and IL-17 could form a pathological axis in SLE, involving T and B lymphocytes, monocytes, DCs and neutrophils, which act in a vicious circle that encourage the preexisting inflammation and propagate the disease process.

Systemic lupus erythematosus (SLE) is an autoimmune disease characterized by heterogeneous clinical manifestations and the presence of multiple cellular and molecular abnormalities in the immune system, including leukocyte activation and cytokine dysregulation. Type I interferons (IFN), and particularly IFNα, are considered to play a central role in SLE etiopathogenesis^1^,^2^. Both IFNα serum levels and expression of IFNα-inducible genes are consistently increased in SLE patients and usually correlate with disease activity and clinical manifestations^3^,^4^,^5^,^6^. Moreover, IFNα from SLE sera can differentiate monocytes into activated dendritic cells (DCs) able to present self-antigens^7^ supporting that this pleiotropic cytokine could be responsible for initiating development of systemic autoimmunity. Binding of IFNα to the two-chain type I interferon receptor (IFNAR) initiates a signal transduction pathways that results in the expression of IFN-induced genes, most of them with immunoregulatory functions on B, T and NK lymphocytes, monocytes/ macrophages, DCs and neutrophils^8^. Consequently, anomalous functioning of type I IFN signalling could be an early event in lupus pathogenesis. As a result, several potential treatments blocking IFNα signalling have emerged in recent years^9^,^10^,^11^.

In the same way, much evidence has highlighted the contribution of B-lymphocyte stimulator (BLyS) to autoantibody production and SLE disease exacerbation^12^. BLyS is produced as a membrane form as well as a soluble protein^12^,^13^ by a wide variety of cells, like B lymphocytes, monocytes, neutrophils and plasmacytoid or myeloid DCs (pDCs and mDCs, respectively)^14^,^15^. Clinical studies have confirmed both circulating and cell surface BLyS overexpression in SLE patients and its correlation with the disease activity^16^,^17^,^18^,^19^.

Previous findings from our group revealed that BLyS expression and mobilization from intra to extra cellular compartments in monocytes can be influenced by IFNα, disease activity or anti-dsDNA levels^19^. Accordingly, there is evidence supporting IFNα as an efficient inducer of BLyS expression. Thus, whereas *in vivo* and *in vitro* IFNα treatment prompts BLyS upregulation^20^,^21^, the therapy with an anti-IFNα monoclonal antibody reduces BLyS expression in SLE patients^22^, suggesting a cooperative action between BLyS and IFNα in the aetiology of SLE. Furthermore, treatment with anti-BLyS monoclonal antibodies in lupus patients was associated with improvements in clinical and laboratory parameters^23^,^24^. However, such clinical trials have not revealed conclusive results since the efficacy of either IFNα or BLyS therapeutic blockade seemed to be dependent on the patients characteristics. Therefore, additional research into the roles played by IFNα and BLyS is required for the identification of SLE patients appropriated for these treatments.

Similarly, IL-17A pathway inhibitors have been recently proposed as a therapeutic option for SLE patients^25^, since increased circulating levels of IL-17 correlated with disease activity and a Th17/Th1 imbalance have been reported in SLE^26^,^27^,^28^. Interestingly, it has been described that IL-17, alone or in synergy with BLyS may stimulate B cell survival and differentiation^29^,^30^,^31^, thus leading to the production of autoantibodies, and consequently IFNα secretion by pDC activated by the resulting immune-complexes^32^,^33^. Indeed, type I IFN has been described to exert a detrimental role in Th17 drive-autoimmune diseases^34^.

Considering previous evidence, the present study aims to evaluate the role of type I IFN signalling in the upregulation of BLyS in SLE patients by analysing the expression of BLyS and IFNRA1 (the α-chain of the common receptor for type-I IFNs) on the membrane of different leukocyte populations, as well as their possible association with the IL-17 production and the serum levels of other relevant pathogenic cytokines.

## Results

### IFNAR1 overexpression on blood leukocytes is associated with membrane BLyS levels

**Figure 1:**
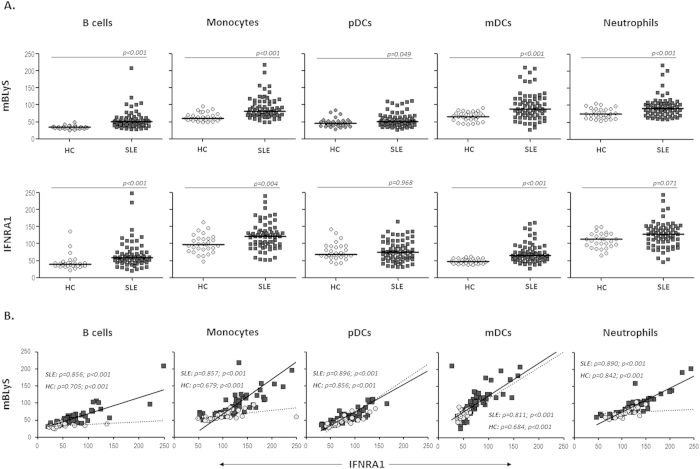
Expression levels of IFNAR1 and membrane BLyS on blood leukocytes from SLE patients and healthy controls. Membrane BLyS (mBLyS) and IFNAR1 levels were quantified by flow cytometry on fresh B cells, neutrophils, monocytes, pDCs and mDCs from 67 patients (SLE) and 29 healthy controls (HC). **(A)** Scatter plots represent MFI levels of BLyS and IFNAR1 in several blood subpopulations and horizontal bars show the median. Statistical significance was assessed by Mann-Whitney U test. **(B)** Graphs show the correlation between mBLyS and IFNRA1 expression (MFI) in each leukocyte population from SLE patients (circles) and healthy controls (squares). Correlation analyses were evaluated by Spearman test.

To extend the knowledge about the previously reported upregulatory *in vitro* effect of IFNα on BLyS induction and release^19^, we wanted to examine the possible relationship between BLyS and IFNAR1 expression on the surface of different circulating fresh cell types. Thus, membrane BLyS (mBLyS) and IFNAR1 levels were quantified by flow cytometry on B cells, neutrophils, monocytes, pDCs and mDCs from SLE patients and healthy controls (HC) (Fig. 1A). As expected, mBLyS expression was upregulated in all studied populations from patients, but especially in neutrophils, monocytes, mDCs and B lymphocytes, the same populations that exhibited higher levels of IFNAR1 compared with HC. Moreover, mBLyS and IFNAR1 levels were closely correlated in all individuals, but more strongly in SLE patients (Fig. 1B).

Multivariate linear regression analysis showed that mBLyS and IFNAR1 upregulation was independent of the treatment, but significant associations were detected with specific clinical manifestations. Thus, mBLyS and IFNAR1 levels on monocytes were related to the frequency of nephritis (β = 0.275, p = 0.032 and β = 0.270, p = 0.027, respectively) and serositis (β = 0.316, p = 0.012 and β = 0.283, p = 0.028), whereas expression on B cells were associated to the frequency of serositis (β = 0.257, p = 0.025 and β = 0.209, p = 0.021) and neurologic disease (β = 0.336, p = 0.005 and β = 0.289, p = 0.017). Therefore, these data indicate an increase in the expression of IFNAR1 in various cell types linked to the pathological widespread overexpression of mBLyS in SLE patients and associated to specific clinical manifestations.

### Circulating BLyS correlated with IFNα and IL-17A serum levels in SLE patients

Next, we analyzed serum levels of BLyS (sBLyS) and IFNα with the aim of determining their possible association with the mBLyS and IFNAR1 overexpression on the different cell types. Interestingly, circulating amounts of sBLyS in SLE patients correlated positively with mBLyS expression on B cells (ρ = 0.273, p = 0.025) and neutrophils (ρ = 0.329, p = 0.007) and the same tendency was observed with IFNAR1 (B cells: ρ = 0.227, p = 0.064; neutrophils: ρ = 0.273, p = 0.026). However, no significant associations were detected between IFNα levels and mBLyS or IFNAR1 expression. Additionally, serum levels of several SLE related cytokines were analyzed in patients and controls (Table 1), showing significant associations of sBLyS with other soluble mediators upregulated in SLE, specifically IFNα, IL-17A, IL-12p70 and MIP-1α (Table 2). Interestingly, in a multiple linear regression model including circulating levels of cytokines, age, gender and disease activity (SLEDAI and anti-dsDNA titer), serum levels of IL-17A remained as an independent predictor of sBLyS (β = 0.252, p = 0.005). Moreover, IL-17A levels correlated with mBLyS expression on neutrophils (ρ = 0.246, p = 0.045), a cell type that is activated by this cytokine in addition to being able to produce it. Therefore, these data suggest a relationship between IFNα, BLyS and IL-17 expression in SLE patients.

### IL-17 producing cells are increased and associated with IFNα levels in SLE patients

**Figure 2:**
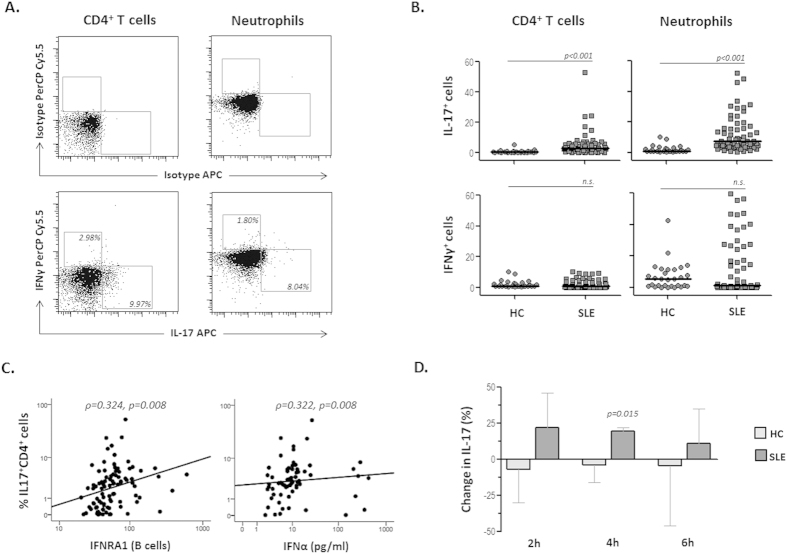
IL-17 producing cells are increased and associated with IFNα in SLE patients. **(A)** IL-17 and IFNγ expression was analyzed intracellularly by flow cytometry in fresh peripheral blood neutrophils and CD4^+^ lymphocytes from SLE patients and HC. Representative dot-plots show positive cells for IL-17 or IFNγ expression, as determined by the fluorescence of cells labelled with the corresponding isotype -matched conjugated monoclonal antibody as a negative control. **(B)** Scatter-plots represent the percentage of IL-17^+^ or IFNγ^+^ cells among CD4^+^ lymphocytes or neutrophils in SLE patients and HC. Horizontal bars show the median. Statistical differences among groups were evaluated by Mann-Whitney U test. **(C)** Relationship between IL-17 producing CD4^+^ T cells and both IFNRA1 and IFNα in SLE patients. Graphs show the proportion of IL-17^+^ CD4^+^ cells related to the IFNRA1 expression on B cells (MFI) and the IFNα serum levels. Statistical significance was evaluated by the Spearman’s rank correlation test. **(D)** IL-17 levels in the culture supernatants of SLE and healthy PBMCs after IFNα treatment. Healthy and SLE PBMCs were treated with IFNα and the release of IL-17 was quantified after 2, 4 or 6 hours of culture. Bars represent median (interquartile range) of the percentage of change in the IL-17 levels in the presence of IFNα compared to unstimulated cultures. Independent experiments were performed with 10 healthy controls and 12 SLE patients. Statistical differences between IFNα**-**treated and untreated cells at each time of culture were evaluated by the Wilcoxon test for paired data. *n.s.*: not statically significant differences.

Therefore, in view of the observed results and since IL-17 can be produced by several cell subsets, including activated Th17 cells and neutrophils^35^, we analyzed the intracellular IL-17 and IFNγ expression in fresh peripheral blood cells from SLE patients and HC (Fig. 2A). The proportion of IL-17^+^ cells was increased among both neutrophils and CD4^+^ lymphocytes from patients compared to controls (Fig. 2B), in accordance with the elevated levels of this cytokine detected in the serum. Remarkably, IL-17^+^CD4^+^ lymphocytes exhibited a positive correlation with IFNα serum levels as well as with IFNRA1 B cell expression in SLE patients, but not in HC (Fig. 2C). These associations were not displayed by IFNγ^+^ cells, thus suggesting that IFNα plays a role in the activation of Th17 cells from SLE patients.

To assess this hypothesis, we performed *in vitro* cultures with PBMCs isolated from SLE patients and HC to analyze the effect of IFNα treatment on IL-17A and BLyS secretion. As was previously reported^19^, IFNα stimulation increased BLyS release in both HC and SLE cultures compared to untreated cells. However, IL-17A secretion was increased in the supernatants of PBMCs cultures from patients but not in those with HC cells (Fig. 2D). Moreover, IL-17A and BLyS levels were positively correlated in SLE cultures (ρ = 0.723, p = 0.031) but not with HC cultures, suggesting an anomalous relationship between BLyS, IFNα and IL-17 in SLE patients.

## Discussion

In recent years, much evidence has demonstrated the crucial role of IFNα and BLyS in the etiopathogenesis of SLE^2^,^36^. Additionally, the upregulation and cellular mobilization of BLyS after IFNα stimulation has been reported^19^,^20^,^21^. Since clinical trials in SLE are conducted with agents that counteract BLyS^23^, its induction by IFNα in patients becomes an important topic. Consequently, the present study extends the proposed relationship between IFNα signaling and BLyS expression in SLE, and reveals a possible pathogenic axis involving both molecules and IL-17 in these patients.

Although prior studies have pointed to a contribution of IFNAR signalling in autoantibody production and renal disease in murine models^9^,^10^,^11^,^37^,^38^, no previous works have analysed the expression of IFNAR in SLE patients. The findings of this study revealed an increased expression of IFNAR1 on various SLE leukocyte populations, which were closely related to the enhanced mBLyS levels. Moreover, both molecules were also correlated in healthy individuals, thus suggesting an intrinsic mechanistic connection between these two pathways.

In agreement with the simultaneous overexpression of IFNAR1 and mBLyS, our cohort of patients showed a positive correlation, not previously reported, between serum levels of IFNα and BLyS. Therefore, it becomes clear that IFNα signalling, via the IFNAR1, could be involved in SLE pathogenesis through the upregulation of BLyS in various cell types, including B cells, monocytes, mDCs and neutrophils. However, only the expression on B cells and neutrophils was positively correlated with the circulating amounts of BLyS.

Another interesting finding of this work was the relationship of IL-17 with both BLyS and IFNα in SLE patients, allowing us to propose a new cytokine pathogenic axis in this disease. IL-17 exerts a proinflammatory effect mediated by the recruitment and activation of monocytes and neutrophils, in addition to the induction of cytokines and chemokines^39^,^40^,^41^. Increased frequency of Th17 cells and elevated amounts of IL-17 have been implicated in the pathogenesis of SLE and other autoimmune diseases^26^,^27^,^28^,^42^,^43^, although the main cellular source of this cytokine in such patients is uncertain. Accordingly, our *ex vivo* assays of blood leukocytes indicated an increased proportion of cells expressing IL-17, not only CD4^+^ lymphocytes but also neutrophils, a population activated in SLE and able to produce this molecule in pathological conditions^44^,^45^. Interestingly, IL-17 serum levels in SLE patients correlated with circulating BLyS as well as with mBLyS expression on neutrophils. In line with this, it has been proposed that BLyS could promote the expansion of Th17 cells^46^, whereas IL-17, alone or in synergy with BLyS may stimulate B cell survival and differentiation^29^,^30^,^31^.

Likewise, IFNα serum levels and IFNRA1 expression on B cells were related to the proportion of CD4^+^ T cells producing IL-17 in patients, suggesting that high IFNα levels, maybe indirectly, could induce IL-17 secretion in SLE. In fact, our *in vitro* experiments support this hypothesis. Whereas our results agree with the observed blocking effect of type I IFNs on the IL-17 production by human PBMCs^47^,^48^,^49^,^50^, IFNα treatment of such cells from SLE patients induced IL-17 secretion, which was positively correlated with BLyS release. In agreement with this, IL-17 has been positively correlated with IFNα and MxA expression, a sensitive marker of type I IFNs activity^51^,^52^, in cutaneous lesions of lupus erythematosus^53^. This effect of IFNα on SLE cells could be due to an a non-canonical IFNAR signalling that can activate the transcription factor STAT3 or the induction of IL-6, both of them critical factors for the Th17 differentiation^54^,^55^,^56^. Moreover, higher percentages of T-helper cells producing IL-17 have been described in SLE patients expressing type I IFN inducible genes, supporting the hypothesis that type I IFN co-acts with Th17 cytokines in SLE pathogenesis^57^.

**Figure 3:**
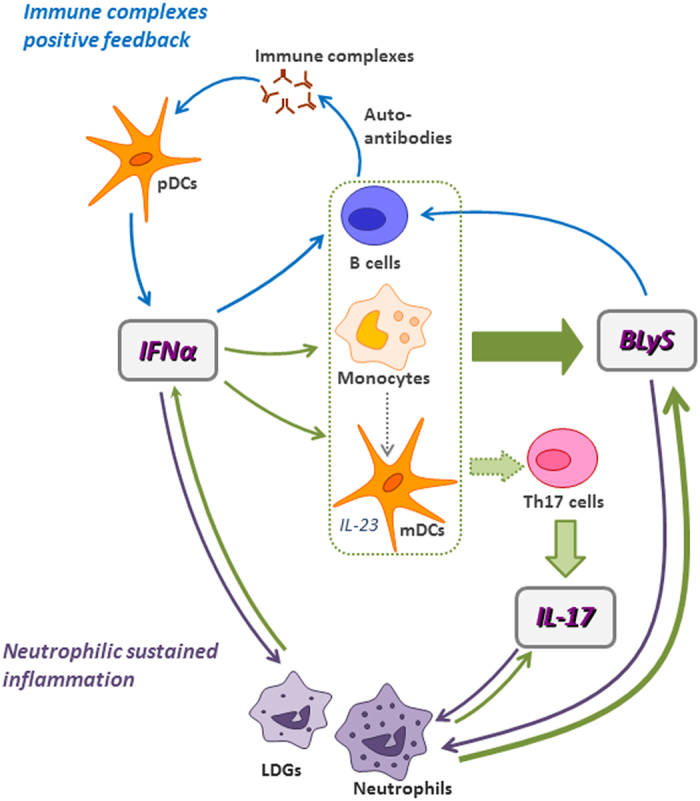
Proposed IFNα, BLyS and IL-17 pathogenic axis in SLE. The high IFNα levels present in most SLE patients could promote the activation and induction of BLyS in B cells, monocytes, mDCs and neutrophils, including the low-density granulocyte (LDG) subset. Thus, the co-stimulation provided by these activated antigen-presenting cells - such as B cells, monocytes or mDCs-, to the Th17 subset enhanced in SLE patients can induce the release of IL-17. Moreover, IL-23 secretion by IFNα-activated mDCs may amplify Th17 differentiation. Also, IFNα-activated neutrophils under the inflammatory conditions presented in SLE are able to secrete IL-17. Then, this vicious circle is closed by the combined effect of IL-17 and BLyS. First, they promoted B cell survival and differentiation, which lead to autoantibody production and further generation of the immune complexes able to induce IFNα secretion by pDCs. In addition, both cytokines can activate neutrophils, thus sustaining the preexisting SLE inflammation. Finally, IFNα secretion by neutrophils, and especially by the LDG subset, propagates the disease process.

In summary, the present study has prompted us to propose the existence of a pathological axis in SLE involving IFNα, BLyS and IL-17, all of which are cytokines with well-known deleterious roles in these patients (Fig. 3). First, the high IFNα levels usually present in SLE patients could be responsible for widespread leukocyte activation and induction of BLyS, as was suggested by the coordinated overexpression of IFNAR1 and BLyS on the membrane of B cells, monocytes, mDCs and neutrophils, and by the positive correlation detected between serum levels of both cytokines. Furthermore, IFNα could promote IL-17 secretion by Th17 cells, increased in SLE patients, probably through the co-stimulation provided by activated antigen-presenting cells, such as B cells, monocytes or mDCs, thus explaining the parallel release of BLyS and IL-17 in SLE cultures after IFNα treatment. Moreover, IFNα-stimulated mDCs secrete IL-23^58^, a cytokine that amplifies Th17 differentiation. Likewise, the altered neutrophils present in SLE patients, over-activated with IFNα, could be also a relevant source of IL-17^59^. Finally, the vicious circle is closed by the effect of IL-17 and BLyS promoting B cell survival and differentiation, which lead to autoantibody production and IFNα secretion by pDCs^32^,^33^. Also, IL-17 and BLyS can activate neutrophils, thus encouraging the preexisting inflammation. Furthermore, SLE neutrophils, and especially the recently described low-density granulocyte subset^60^,^61^, could secrete increased levels of IFNα and form neutrophil extracellular traps (NETs), which in turn would lead to more IFNα synthesis by pDCs leading to a self-perpetuating cycle. Therefore, the concomitant participation of IFNα, BLyS and IL-17 in a pathogenic axis could be a key factor underlying the incomplete response to therapies based on the blockade of these cytokines in a subset of SLE patients. Hence, given the heterogeneity of the disease, determination of the individual cytokine profile could be useful for the identification of SLE patients candidates for biological therapies blocking such targets, alone or in combination.

## Methods

### Patients and controls

Patients included in the study were not hospitalized individuals fulfilling at least four American College of Rheumatology (ACR) revised criteria for the SLE classification^62^, and that were sequentially recruited from the outpatient clinic of the Autoimmune Disease Unit (Hospital Universitario Central de Asturias, HUCA). Information on clinical features during the disease course was obtained after a retrospective review of clinical histories. At the time of sampling, patients were asked precise questions regarding the treatment received over the previous 3 months and anti-dsDNA titer and SLE disease activity index (SLEDAI) were determined. *Ex vivo* cytometric analysis were performed in sixty-seven patients (Table 3) and twenty-nine healthy controls (mean age ± SD: 47.73 ± 6.56 years; female/male: 21/8) recruited from the same population. For cytokine quantification, additional serum samples from 199 SLE patients (Supplementary Table S1) and 90 sex and age-matched healthy controls were collected (mean age ± SD: 45.37 ± 10.31 years; female/male: 78/21), including those previously recruited for cytometry analysis.

### Ethics approval

Ethical approval for this study was obtained from the Regional Ethics Committee for Clinical Research (Servicio de Salud del Principado de Asturias), according to the Declaration of Helsinki. Written informed consent was signed by all individuals prior to participation in the study. All methods were carried out in accordance with the approved guidelines.

### Flow cytometry analysis

Peripheral blood was collected with EDTA as anticoagulant. BLyS and IFNRA1 levels were simultaneously quantified by flow cytometry on B cells, neutrophils, monocytes, pDCs and mDCs, whereas IL-17 and IFNγ positive cells were identified among CD4^+^ lymphocytes and neutrophils. Monoclonal antibodies specific for IFNRA1 (PE) (R&D Systems, USA), CD19 (CF-Blue) (Immunostep, Spain), CD14 (APC-Cy7) (BioLegend, USA), CD303 (BDCA-2) (PECy7), BLyS (FITC), CD123 (PerCP-Cy5.5), CD1c (BDCA-1) (APC), CD4 (APC-Cy7), IL-17 (APC), IFNγ (PerCP-Cy5.5) and isotype, concentration and fluorochrome-matched control antibodies (all from eBioscence, USA) were employed for the cytometry analysis. The specificity of the monoclonal antibody for BLyS (clone 1D6)^63^ was confirmed in B lymphocytes and peripheral blood mononuclear cells after incubation with 10 or 100 ng/ml of soluble BLyS (eBioscience) at different times (0, 10, 30 and 60 minutes) followed by anti-BLyS (clone 1D6) staining. Blood cells were stained extracellularly with the appropriate monoclonal antibody to identify all the subpopulations for 30 min at 4°C and then cells were washed twice in staining buffer and resuspended in PBS. To determine IL-17 and IFNγ positive leukocytes, blood cells were fixed, permeabilized and intracellularly stained with monoclonal antibodies against these cytokines following the manufacturer’s instructions (Fixation/permeabilization buffer set; eBiosciences). Acquisition of 200,000 events and 10,000 CD4^+^ cells/tube was performed using a FACSCanto II flow cytometer (BD Biosciences). The analysis was based on cells located in an area of plots termed “the living region” which was defined using forward and side scatter. Then, different leukocyte subpopulations were identified according to the expression of specific surface markers, as previously described^19^. Thus, pDCs were identified as double positive cells for the expression of BDCA-2 and CD123, mDCs were defined as CD19^−^ BDCA-1^+^, while monocytes, CD4^+^ lymphocytes and B cells and were identified by expression of CD14, CD4 or CD19, respectively. Neutrophils were identified according to their distinctive forward and side-scatter signal. Supplementary Table S2 shows the number of events counted in the different cellular subpopulations. Samples were subsequently analyzed using FlowJo software (Scripps Research Institute, San Diego, CA). Results were expressed as the percentage of positive cells or mean fluorescence intensity (MFI) of gated populations after subtracting the fluorescence of the background of the respective isotype control from the total fluorescence.

### *In vitro* cultures

Peripheral blood mononuclear cells (PBMC) from healthy donors and patients were obtained by centrifugation over Ficoll-Hypaque gradients (Lymphoprep, Nycomed). PBMCs, at a density of 2 × 10^6^/ml, were cultured in complete RPMI medium (RPMI 1640 containing 2 mM L-glutamine and 25 mM Hepes, supplemented with 10% heat-inactivated fetal calf serum and the antibiotic streptomycin and ampicillin at 100 μg/ml) at 37 °C and 5% carbon dioxide in presence or absence of human IFNα2b (Intron-A, 1000 U/ml). At different times of culture (2, 4 and 6 hours), cell-free supernatants from these cultures were collected for IL-17A and BLyS quantification.

### Cytokine quantification

Culture supernatants and serum samples from SLE patients and HC were maintained at −80°C until cytokine determinations. IFNα, IL-10, IL-12p70, IL-1β, IL-17A, MIP-1α (CCL3) and GM-CSF amounts were quantified by Cytometric Bead Arrays Flex Set in a BD FACS Canto II flow cytometer (both from BD). For IL-1β and IL-10, an Enhanced Sensitivity Flex Set was needed. ELISA kits were used for the quantification of TNFα (Mini EDK kit, PeproTech), IFNγ (OptEIA kit, BD) and BLyS (Human BAFF Instant ELISA, eBioscience) following the manufacturer’s instructions. The lower limits of detection were 1.25 pg/ml for IFNα, 13.7 fg/ml for IL-10, 0.6 pg/ml for IL-12p70, 48.4 fg/ml for IL-1β, 0.3 pg/ml for IL-17A, 0.2 pg/ml for MIP-1α, 0.2 pg/ml for GM-CSF, 3.9 pg/ml for TNFα, 0.58 pg/ml for IFNγ and 0.13 ng/ml for BLyS.

### Statistical analysis

The Kolmogorov-Smirnov test was used to assess the normal distribution of the data and nonparametric testing was used to determine differences between patient and control groups (Mann-Whitney U-test), while correlations were examined by Spearman’s rank correlation test. Multivariate linear regression analyses including treatments, demographic and clinical parameters were performed to determine the influence on the mBLyS and IFNRA1 expression. Variables were log-transformed to achieve normal distribution and standardized linear regression coefficients (beta) were used as an estimate of the association. Data were expressed as the median (interquartile range). A p-value < 0.05 was considered statistically significant. Data were analysed using GraphPad Prism 5 software (GraphPad Software, USA) and SPSS 22 statistical software package (SPSS Inc.).

## Additional Information

**How to cite this article**: López, P. *et al.* A pathogenic IFNα, BLyS and IL-17 axis in Systemic Lupus Erythematosus patients. *Sci. Rep.* **6**, 20651; doi: 10.1038/srep20651 (2016).

## Supplementary Material

Supplementary Information

## Figures and Tables

**Table 1:** Serum levels of cytokines in SLE patients and healthy controls.

Cytokines	HD (n = 90)	SLE (n = 199)	p-value
BLyS	648.46 (390.22–1067.41)	1430.46 (823.28–2052.51)	<0.001
IFNα	1.39 (1.25–2.09)	5.81 (2.52–12.38)	<0.001
IL-17A	3.37 (0.96–4.10)	3.87 (1.44–12.24)	0.037
IL-1β	0.80 (0.60–1.23)	1.09 (0.88–1.45)	<0.001
IFNγ	3.06 (2.08–6.68)	3.22 (2.21–4.90)	0.819
TNFα	93.94 (6.63–209.51)	154.23 (91.71–229.39)	0.006
IL-10	0.28 (0.02–0.91)	1.39 (0.98–2.01)	<0.001
GM-CSF	1.00 (0.30–2.39)	2.22 (0.30–4.87)	0.001
IL-12p70	1.43 (1.22–1.67)	1.65 (1.34–1.92)	0.001
MIP-1α	0.20 (0.20–4.48)	5.70 (0.37–11.46)	<0.001

Values represent median (interquartile range) (pg/ml). Differences between SLE and HC were evaluated by Mann-Whitney U test.

**Table 2:** Serum BLyS association to other circulating cytokines in SLE patients.

Cytokines	Correlations with sBLyS	
	Correlation coefficient	p-value
IFNα	0.249	<0.001
IL-17A	0.245	0.001
IL-1β	0.107	0.135
IFNγ	−0.016	0.831
TNFα	0.141	0.119
IL-10	0.109	0.128
GM-CSF	0.162	0.072
IL-12p70	0.152	0.032
MIP-1α	0.155	0.031
Correlation analyses were evaluated by Spearman test.		

**Table 3:** Demographic and clinical features of SLE patients.

Total SLE patients (n)	67
Sex (female/male) (n)	63/4
Age, years (mean ± SD)	49.05 ± 11.48
Age at diagnosis, years (mean ± SD)	34.69 ± 13.22
Disease duration, years (mean ± SD)	14.01 ± 10.25
SLEDAI score^b^ [median (IQR)]	3 (0–6)
Clinical manifestations^a^, n (%)	
Malar rash	34 (50.75)
Discoid lesions	14 (20.90)
Photosensitivity	34 (50.75)
Oral ulcers	36 (53.73)
Arthritis	48 (71.64)
Serositis	13 (19.40)
Cytopenia	44 (65.67)
Renal disorder	15 (22.39)
Neurological disorder	7 (10.45)
Autoantibodies^a^, n (%)	
ANAs	67 (100.00)
Anti-dsDNA/titer^b^, U/ml (mean ± SD)	53 (79.10)/32.13 ± 49.38
Anti-SSA/Ro60	27 (40.30)
Anti-Ro52/TRIM21	23 (34.33)
Anti-SSB	11 (16.42)
Anti-Sm	5 (7.46)
Anti-U1RNP	10 (14.92)
RF	11 (16.42)
Anti-RibP	7 (10.45)
Treatment^b^, n (%)	
None or NSAIDs	2 (2.98)
Antimalarial drugs	58 (93.55)
Glucocorticoids/dose, mg/kg [median (IQR)]	27 (43.55)/7.50 (5.00–10.00)
Immunosuppressants (mycophenolate mophetil)	1 (1.61)

SD: standard deviation; IQR: interquartile range; NSAID: non-steroidal anti-inflammatory drug; anti-RibP: anti-ribosomal-P; anti-dsDNA: anti-double-stranded DNA; TRIM21: tripartite motif-containing protein 21.

^a^Cumulatively registered.

^b^At time sampling.
