# Supplementary material for: A pathogenic IFNα, BLyS and IL-17 axis in Systemic Lupus Erythematosus patients
Source: Sci Rep. 2016 Feb 5;6:20651. doi: 10.1038/srep20651 (PMC4742957; doi:10.1038/srep20651)
Supplement: Supplementary Information [file srep20651-s1.pdf]

SUPLEMENTARY DATA

Title: A pathogenic IFNα, BLyS and IL-17 axis in Systemic Lupus Erythematosus patients.

Patricia Lopez, Javier Rodríguez-Carrio, Luis Caminal-Montero, Lourdes Mozo, and Ana Suarez.

Supplementary Table S1. Demographic and clinical features of SLE patients

**Total SLE patients (n)**	**199**
Sex (female/male) (n)	189/10
Age, years (mean ± SD)	48.27 ± 13.81
Age at diagnosis, years (mean ± SD)	35.41 ± 13.33
Disease duration, years (mean ± SD)	12.23 ± 9.12
SLEDAI score[b] [(median (IQR)]	4 (2-6)
Clinical manifestations[a], n (%)	
Malar rash	103 (51.76)
Discoid lesions	42 (21.11)
Photosensitivity	112 (56.28)
Oral ulcers	100 (50.25)
Arthritis	140 (70.35)
Serositis	39 (19.60)
Cytopenia	133 (63.83)
Renal disorder	60 (30.15)
Neurological disorder	17 (8.54)
Autoantibodies[a], n (%)	
ANAs	199 (100.00)
Anti-dsDNA / titer[b], U/ml (mean ± SD)	127 (63.82)/38.66 ± 65.84
Anti-SSA/Ro60	81 (40.70)
Anti-Ro52/TRIM21	63 (31.66)
Anti-SSB	31 (15.58)
Anti-Sm	20 (10.05)
Anti-U1RNP	28 (14.07)
RF	30 (15.07)
Anti-RibP	27 (13.57)
Treatment[b], n (%)	
None or NSAIDs	19 (9.55)
Antimalarial drugs	159 (79.90)
Glucocorticoids / dose, mg/kg [median (IQR)]	79 (39.70) / 7.50 (5.00-10.00)
Immunosuppressants[c]	21 (10.55)

[a]Cumulatively registered
[b]At time sampling
[c]Methotrexate, azathioprine, cyclophosphamide, cyclosporine A or mycophenolate mophetil.
SD: standard deviation; IQR: interquartile range; NSAID: non-steroidal anti-inflammatory drug. anti-RibP: anti-ribosomal-P; anti-dsDNA: anti-double-stranded DNA; TRIM21: tripartite motif-containing protein 21.

Supplementary Table S2. Number of cells analysed in the different cellular populations of SLE and HC.

Cellular populations	HC (N=29)	SLE (N=67)
**Monocytes**	13,500 (9881-14300)	13,200 (10,900-19,850)
**pDCs**	548 (427-759)	429 (285-659)
**mDCs**	722 (624-926)	447 (275-597)
**B cells**	10,600 (7,968-11,700)	6,605 (3,897-11,400)
**Neutrophils**	140,000 (123,000-167,000)	207000 (143,500-290,500)

Data represent median (interquartile range).
